# Using Theories of Change to inform implementation of health systems research and innovation: experiences of Future Health Systems consortium partners in Bangladesh, India and Uganda

**DOI:** 10.1186/s12961-017-0272-y

**Published:** 2017-12-28

**Authors:** Ligia Paina, Annie Wilkinson, Moses Tetui, Elizabeth Ekirapa-Kiracho, Debjani Barman, Tanvir Ahmed, Shehrin Shaila Mahmood, Gerry Bloom, Jeff Knezovich, Asha George, Sara Bennett

**Affiliations:** 1 Department of International Health, Johns Hopkins Bloomberg School of Public Health, 615 N. Wolfe Street, Baltimore, MD 21205 United States of America; 2 Institute of Development Studies, Library Road, Brighton, BN1 9RE United Kingdom; 3 Department of Health Policy, Planning and Management, Makerere University School of Public Health, New Mulago Hospital Complex, Kampala, Uganda; 4 Epidemiology and Global Health Unit, Department of Public Health and Clinical Medicine, Umeå University, 901 87 Umeå, Sweden; 5 IIHMR University, 1 Prabhu Dayal Marg, Sanganer, Jaipur, 302029 India; 6 Health System and Population Studies Division, ICDDR,B, 68 Shaheed Tajuddin Ahmed Sarani, Mohakhali, Dhaka, 1212 Bangladesh; 7 Quaternary Consulting, London, United Kingdom; 8 School of Public Health, University of the Western Cape, Cape Town, South Africa

**Keywords:** Theory of change, Learning by doing, Bangladesh, India, Uganda

## Abstract

**Background:**

The Theory of Change (ToC) is a management and evaluation tool supporting critical thinking in the design, implementation and evaluation of development programmes. We document the experience of Future Health Systems (FHS) Consortium research teams in Bangladesh, India and Uganda with using ToC. We seek to understand how and why ToCs were applied and to clarify how they facilitate the implementation of iterative intervention designs and stakeholder engagement in health systems research and strengthening.

**Methods:**

This paper combines literature on ToC, with a summary of reflections by FHS research members on the motivation, development, revision and use of the ToC, as well as on the benefits and challenges of the process. We describe three FHS teams’ experiences along four potential uses of ToCs, namely planning, communication, learning and accountability.

**Results:**

The three teams developed ToCs for planning and evaluation purposes as required for their initial plans for FHS in 2011 and revised them half-way through the project, based on assumptions informed by and adjusted through the teams’ experiences during the previous 2 years of implementation. All teams found that the revised ToCs and their accompanying narratives recognised greater feedback among intervention components and among key stakeholders. The ToC development and revision fostered channels for both internal and external communication, among research team members and with key stakeholders, respectively. The process of revising the ToCs challenged the teams’ initial assumptions based on new evidence and experience. In contrast, the ToCs were only minimally used for accountability purposes.

**Conclusions:**

The ToC development and revision process helped FHS research teams, and occasionally key local stakeholders, to reflect on and make their assumptions and mental models about their respective interventions explicit. Other projects using the ToC should allow time for revising and reflecting upon the ToCs, to recognise and document the adaptive nature of health systems, and to foster the time, space and flexibility that health systems strengthening programmes must have to learn from implementation and stakeholder engagement.

**Electronic supplementary material:**

The online version of this article (doi:10.1186/s12961-017-0272-y) contains supplementary material, which is available to authorized users.

## Background

In the broader discussion around how to improve the implementation of development programmes and how to learn from implementation, there is a rapid growth of interest in the Theory of Change (ToC) tool, particularly in the health sector. For the purpose of this paper, we define the ToC as a management tool, “*an outcomes-based approach which applies critical thinking to the design, implementation, and evaluation of initiatives and programs intended to support change in their context*” [1]. Generally, ToC development includes an analysis of how an intervention created change in a particular area, a description of the pathways through which this change is expected to happen, and a framework for testing whether and how change happens [2]. The ToCs usually have two components, a visual depiction, for example, of key variables, stakeholders and pathways of change, and a related narrative account [2]. The ToC narrative generally includes information about the context in which an intervention is implemented (including social, political and environmental conditions), the current state of the problem, the actors to influence change, an outcome of desired long-term change, a description of process/sequence of change, and the underlying assumptions [1].

The development and evaluation literature describes the ToC tool in various ways, without consensus [2]. In the evaluation literature, ToCs were developed as an extension of the logic planning models, like the logframe [3]. In the context of the social practice literature, ToCs arose from the desire of project implementers to systematically and “*consciously reflect on the underlying theories for development practice*” [3]. As such, ToCs are most frequently used to illustrate and test assumptions and hypothesised pathways of change [4–12], to guide data collection [13], or to explain impact or changes discovered through the final evaluation [3, 14–16]. Indeed, recent interest in ToCs can be partially explained as a response to the inflexibility in logframes. Historically, logframes have been frequently used in international development, yet, due to their linear and often rigid form, make it difficult “*to analyze* [the] *messy social processes*” that are common in this field [3]. By contrast, ToCs allow for a detailed explanation of assumptions and pathways of change and are increasingly perceived as ‘living documents’, amenable to incorporating new knowledge and assumptions, as well as unintended consequences in implementation, and updated ToCs may guide a final evaluation [1, 3].

The ToC is also often recognised as a tool for “*ToC thinking*” [2], iterative design and implementation [17, 18] and, preferably, is participatory in nature [16, 19–23]. However, in most cases, implementers develop ToCs during the initial design of a project, often on their own or in collaboration with evaluators, but only as a one-off exercise and are referred to again for the final evaluation. ToCs might be typically revisited over the course of programme implementation, but revisions are very seldom documented. In general, ToCs are re-emerging as an essential component of specific evaluation approaches, such as realist evaluation [24, 25], or efforts to foster a “*whole-system view*” [26]. Furthermore, the ToC has become a step in the intervention design process that some funding agencies require. DfID, for example, has been formally working with ToC since 2010 as part of a broader effort in DfID to enhance the evaluation of their programmes [1].

A review by Stein and Valters [2] identified four broad categories for how ToCs can be used, namely strategic planning, monitoring and evaluation, description, and learning. When applying their framework to examining how ToCs are used in day-to-day practice in the context of the Asia Foundation’s work, Valters et al. [3] found that ToCs were primarily used for communication, learning and accountability purposes. The process of ToC development combined logical thinking and critical reflection, both to map the pathways from inputs to outcomes, as well as to lead a team through a “*deeper reflective process and dialogue*” to “*deconstruct the basic assumptions which underpin program interventions*” [3]. Besides the analysis by Valters et al. [3], the recent uses and applications of ToCs in this “*deeper reflective process*” have not been often documented, particularly in low- and middle-income countries [1]. Indeed, a more recent paper by Valters [27] warned that, without an emphasis on process, ToCs risked becoming a management tool that failed to foster the desired learning and reflection. As such, this paper’s documentation of the ToC revision process, an activity largely absent from the literature, will strengthen the understanding of how ToCs can achieve their potential.

Future Health Systems (FHS) is a DfID-funded Research Programme Consortium focused on providing knowledge about how health systems can improve the quality of and access to basic health services for poor and socially marginalised people in diverse health systems contexts. As required in the funder’s request for proposals, FHS developed ToCs in its second phase of implementation (2011–2016). In this paper, we primarily document the process through which the FHS consortium country teams from Bangladesh, India and Uganda used ToCs in their research activities. We seek to reflect on the FHS experience and, where possible, shed light on some of the outstanding debates about ToCs, inclduing when should ToCs be developed, whether and how they should be revised, what level of evidence is needed to develop a ToC, and when and how to incorporate stakeholders’ perspectives.

## Methods

All five countries participating in the FHS consortium – Afghanistan, Bangladesh, China, India and Uganda – developed ToCs in 2011. At the recommendation of the Consortium Advisory Group, the FHS teams from Bangladesh, India and Uganda revised their ToC about half-way through project implementation, between late 2013 and mid-2014. ToCs were not revised for Afghanistan (where FHS activities were scheduled to end early) or China (where the FHS team was studying an intervention over which it had no direct control as it was managed by the Ministry of Public Health). This article summarises reflections from the three teams (Bangladesh, India and Uganda) that had experience with both the development and revision of ToCs.

At the project’s annual meeting in June 2014, country team representatives from Bangladesh, India and Uganda, as well as colleagues from Johns Hopkins Bloomberg School of Public Health and the Institute of Development Studies held an initial meeting and brainstorming to discuss how to best reflect upon and share their experience with ToCs. The large majority of reflection meeting participants had several years of experience within the FHS project (either as part of the management or country research teams) and included key individuals who were part of the initial design of the project and the ToC.

After the June 2014 meeting, members of the FHS consortium engaged in a reflection on the ToC process, guided by a series of questions on the motivation, development, revision and use of ToCs, as well as on the benefits and challenges of the process. Where it was not possible to obtain information by e-mail, LP, AW and JK followed-up with Skype or in-person discussions. A final reflection meeting was held in June 2015, specifically to understand whether and how the country teams were planning to use the ToC in the final year and a half of the project, including as part of their final monitoring and evaluation activities. We explore the FHS consortium’s experience using ToCs across the four main ToC purposes that Valters et al.’s outline – strategic planning, communication, learning and accountability [2, 3, 27]. Though we present them as discrete, they represent broad categories, which overlap in practice [2].

## Results

In this section, we document how the FHS consortium came to revise the ToCs, and reflect on each team’s use of ToCs in relation to strategic planning, communication, learning and accountability [2, 3, 27].

As mentioned above, the teams revised their ToCs about half-way through the project, at the recommendation of the Consortium Advisory Group. Revising the ToCs provided the project teams with an opportunity to reflect on lessons learned from the first 2 years of implementation. Box 1 summarises the main research topics and interventions for the three country teams.

**Box 1** Summary of the FHS teams’ initial intervention foci

**Table:** 

The Bangladesh team aimed to strengthen the linkages between informal healthcare providers, i.e. village doctors, and the formal health system in Chakaria through eHealth, especially through mHealth and telemedicine.
The India team worked in the Sundarbans areas that have poor health indicators, in addition to being vulnerable to climate shocks. The team focused on filling information gaps and improving the coordination of service providers, in order to foster evidence-led decision-making and to improve overall child health outcomes.
The Uganda team used a participatory action research approach to build more sustainable financing mechanisms to increase access to skilled delivery for women in rural Uganda. The main intervention components included health systems strengthening, community sensitisation home visits by village health teams, radio spots, community dialogues, and promotion of saving practices for birth preparedness.

The teams’ initial and revised ToC figures (as well as the overall project ToC) are found in Additional file 1. These were also accompanied by narratives that detailed assumptions and descriptions of the pathways through which change was hypothesised (narratives available by request). The nature and the extent of the revisions varied by country. For example, in Bangladesh, the major change in the ToC related to a change in the actual intervention. After HealthBox – the team’s platform providing community members and village doctors with self-diagnosis and treatment guide information – was dropped due to technical reasons, the Bangladesh team re-focused their intervention on telemedicine only. In India, the most significant change highlighted through the ToC revision was the transition from a child health-focused theory to one that focused more broadly on maternal and child health stakeholders and outcomes, recognising the broader linkages between health, nutrition, livelihood and climate change. Additionally, the revised ToC included more refined assumptions around non-state actors, the presence and actions of donor agencies, the political environment, as well as the risk of recurrent climatic shock. In Uganda, the revised ToC placed more emphasis on illustrating the central role of participatory action research (PAR) and how it brings together various stakeholders [28, 29]. Overall, all of the revised ToCs exhibited far greater feedback among intervention components, as well as among the actors involved, and the possible outcomes and impact measures. Table 1 presents further details about the ToC development and revision process for each country.

**Table 1:** Summary of the country projects and the process of developing and revising the ToC

	Bangladesh	India	Uganda
ToC development	Actors: Bangladesh FHS team, PIRU Coordinator, TRCL representatives Duration: 2–3 months Materials: Document review, team meetings, discussion with external collaborators and insights from other FHS members	Actors: India FHS team, PIRU Coordinator, facilitation by country coordinator Duration: 2–3 months Materials: Team meetings during project design and developing of annual plan and status update against the annual plan	Actors: Uganda FHS team, facilitation by EE, Suzanne Kiwanuka, MT and JK Duration: 9 months Materials: Stakeholder consultations during project design phase
ToC revision	Actors: Bangladesh FHS team Duration: 1-day workshop Materials: data from household survey, interviews with village doctors, patients, project documents	Actors: India FHS team, facilitation by PIRU Coordinator Duration: 1-day workshop Materials: Findings from internal evaluation of implementation challenges; in-depth interviews with various stakeholders like non-governmental organisation, donor agencies and government workers and officials	Actors: Uganda FHS team, facilitation by AG and LP Duration: 2-day workshop Materials: PAR cycles allowed for periodic review of intervention; quarterly meetings at the sub-county and district levels and community engagement informed the ToC revision
Key changes made	Revised intervention (dropped HealthBox, focused on telemedicine only); increased emphasis on inputs such as promotional activities by the telemedicine providers; identified new linkages, such as between (1) community and telemedicine use and (2) telemedicine use by the poor playing a role in reducing the delay in care-seeking	Constructs and relationships more specific, particularly to better recognise health and non-health factors influencing child health, as well as of historical and political contextual factors affecting the team’s intervention with feasible indicators	Richer representation of the complex nature of the project’s interventions (i.e. greater representation of feedback among intervention components and among stakeholders); assumptions better articulated in the revised ToCs
Major contextual changes captured	Rapidly growing mobile phone subscriptions were assumed to facilitate access to and use of eHealth initiatives by the community and village doctors; however, use of eHealth services by the community and village doctors appears to be very limited	The local stakeholders in Sundarbans were not working solely on child health; rather, they employed an approach cutting across health, nutrition, livelihood and climate change	Changing in the channels of communicating messages to the communities, the content of the messages, as well as re-targeting actors responsible for various interventions at district level

*Abbreviations*: *FHS* Future Health Systems, *PIRU* Policy Influence Research Uptake, *TRCL* Telemedicine Reference Center Ltd., Bangladesh, *PAR* participatory action research

### Strategic planning

The FHS teams’ development of their ToCs in 2011 followed DFID guidelines to Research Partner Consortia, which required the use of this tool in the “*thinking, planning, and evaluation components of the programme process*” [2]. A consortium-wide ToC was developed alongside the consortium’s logframe. Given the decentralised structure of the consortium and the uniqueness of country research studies, the consortium decided that it was more appropriate for all teams to develop country-specific ToCs. Therefore, across FHS teams, the ToCs were developed as a means to further justify and expand on the consortium’s ‘assumptions’ proposed in the logframe and how they changed, particularly to account for the local contexts in which activities were implemented [2]. For all countries, the ToCs were developed in both a visual and narrative way, as the diagrams alone were not always intuitive without their accompanying narrative.

Later on, and especially through the ToC revision process, the country teams shifted how they used the ToC, to exhibit more of the “*ongoing critical reflection on both the specific (changing) context and how programme rationales and strategies fit into this*” [27]. In order to minimise repetition, we describe how the specific consortium teams used the ToCs throughout the process of implementation in the subsequent sections. In summary, while there was less strategic use of the ToCs at the consortium-level, where logframes were more appropriate, the ToCs played an important role in the design phase of the three country research studies. Nonetheless, the ToC revision seems to have had less of a strategic impact on the direction of projects as it focused primarily on adapting the ToCs to document each team’s lessons from early implementation.

### Communication

Valters [3] mentions two ways through which ToCs can facilitate communication, namely (1) communicating internally, to staff within the organisation, regarding assumptions and goals, and (2) communicating externally with donors, partners (i.e. presenting the research programme coherently and concisely – a story) and governments. In addition, a third possible communication dimension was particular to the FHS interventions planned, namely the communications activities embedded within the projects themselves, particularly as several of the country-level activities had strong education and behaviour change communication components.

### Internal and cross-team communication

In terms of internal cross-team communication, the ToC development revision processes gave all three teams the opportunity to engage in brainstorming and consensus-building around the main pathways of change, related assumptions and contextual elements, as well as how these evolved during the implementation process. This was particularly important for the India team, which included a number of new members of staff when the project was initially designed. The initial ToCs also proved useful for communication across the various FHS country teams. They were presented at the annual FHS consortium-wide meeting in Uganda in 2011 and allowed the various teams to understand what sort of research activities were being undertaken in each location. Although each ToC was designed to be relevant at country-level, in seeing them presented together, the entire consortium team gained better insights into the uniqueness and value added of each country intervention and of the project as a whole. With an explicit understanding of how each of the teams intended to proceed with their research, the FHS management team constructed relevant support processes, logframes and other materials. For example, the FHS management team identified some of the capacity development activities undertaken by the overall consortium through discussions around the country-level ToCs. The Consortium Advisory Group suggestion that the ToCs be revised mid-project is also evidence of the usefulness of ToCs for facilitating internal communications. The mid-project revision process was introspective, and used primarily for communication within each research team, and between country teams and the overall consortium management.

### External communication

In Bangladesh, the ToC helped in two ways – (1) guiding the research team through discussions among various stakeholders including policy-makers and (2) as a facilitator for engaging the community in a more meaningful and participatory way for focused and open dialogues, particularly to understand the underlying assumptions related to the course of interventions/projects. In Bangladesh, representatives from Telemedicine Reference Center Ltd. (TRCL), the private firm that the team was seeking to engage, were included in the initial ToC development workshop. The revision was made during the course of an internal workshop organised to reflect, refocus and redesign, in which the entire Bangladesh research team participated. No external stakeholders were involved in the revision process and the revised ToC has not been shared outside of the project.

As part of their knowledge intervention, the India team catalysed the Sundarbans Learning Platform, which served to bring together key actors engaged in child health service delivery. External stakeholders were not directly involved in the ToC revision process. Instead, the research team interacted with various stakeholders during the project implementation and heeded their advice to consider child health issues in a broader context of livelihood, health and nutrition.

In Uganda, the development and revision helped the team to think about which stakeholders must be engaged and the subsequent changes the team envisioned in stakeholders’ attitudes and practices. As the Uganda team adopted a PAR approach, they captured stakeholders’ views systematically, but separate from the ToC development and revisions processes, which were not communicated directly beyond the research team. Knowledge gained through local stakeholder consultations (i.e. with health workers, the district health teams, the political and administrative leaders of the districts, religious and opinion leaders, and local implementing partners) informed the ToC processes and helped the Ugandan team to target their communications to specific stakeholder groups in both the initial and revised ToCs. For example, about half-way through the implementation of their interventions, the project team wanted to emphasise the importance of male involvement, e.g. men escort their wives to health facilities for antenatal care and delivery and households join savings groups. The team introduced a new intervention component consisting of radio spot messages and held meetings with various stakeholder groups, such as district councils or the Ministry of Health’s Maternal and Child Health Cluster, and communicated to them the specific actions that arose out of the pathways specified in the ToC.

### Planning effective research communication

When it came to developing communication activities, the ToC process helped to identify specific ways in which research communication could support the desired outcomes during implementation. In India, for example, external stakeholders were not engaged directly in the ToC development process. However, the ToC-related discussions guided the project’s different communication channels and emphasised the need for a shared knowledge platform (i.e. the Sundarbans Learning Platform) of the various actors working on health in the Sundarbans. In Bangladesh and Uganda, while the ToC diagram and narrative itself were not communicated directly with stakeholders, they served as the research teams’ guide for the development of videos, newsletters, evidence briefs, journal publications and policy briefs.

### Learning

Valters argues that the ToC process is supposed to facilitate ‘double-loop’ rather than ‘single-loop’ organisational learning [3]. ToCs should provide a platform for teams to make ‘critical reflections’ that question the underlying organisational goals, values and rules (i.e. ‘double-loop learning’), rather than adjusting strategies within the existing organisational framework (i.e. ‘single-loop learning’) [3, 30]. In order for learning to be encouraged, the initial assumptions must be clearly defined. Valters cautions, however, that this can be challenging – the assumptions outlined in a ToC can be unclear, not sufficiently problematised, based on weak and possibly selective evidence [3].

All of the FHS teams confirmed that the process of defining assumptions prior to implementation, during the initial ToC development, was challenging. Consequently, the teams identified much of the learning from the ToC through the process of revising the ToCs, when assumptions were revisited, challenged and revised based on new evidence and experiences arising from the first 2 years of implementation.

Overall, across all three countries, the first ToCs could not initially account for all the system’s complexities and the project teams could not anticipate all of the contextual changes which were to ensue over the first 2 years of implementation. Similarly, all of the country teams reported some degree of learning through the revision process. We therefore take a closer look at the learning process and key lessons learned by each country team.

### Bangladesh

In Bangladesh, the first ToC outlined an intervention that was very ambitious, focusing on two activities of engaging with and strengthening the capacity of informal providers, namely the HealthBox and telemedicine, implemented through a United States-based research agency called RTI International and a partnership with TRCL, respectively. As both TRCL and RTI International eventually withdrew from the programme, the research team had to revisit the intervention package and its underlying assumptions for change, including their reliance on external partners. Consequently, both HealthBox and telemedicine were dropped from the intervention package. In light of these major shifts and unfounded assumptions about partner commitment and capacity, the process of revising the ToC facilitated the synthesis and generation of learning within the team, providing an opportunity to discuss progress and review new evidence about intervention outcomes and context. For example, as part of gathering more evidence in regards to the pathways outlined in the initial ToC, the research team conducted a community survey at the end of 2012 on access to mHealth services and the burden of disease [31, 32]. Initial results from this survey suggested low penetration of telemedicine services, despite high mobile phone coverage. Only 5% of the community members were aware of the mHealth services and, among them, only 11.6% used the services. The community also expressed mixed views on the quality of services, for example, while the speed and convenience of telemedicine was noted, respondents were reluctant to sacrifice the perceived benefits of face to face consultations [31–33]. This evidence was in conflict with the team’s initial assumptions, because, despite many mHealth interventions being implemented, community awareness and use of related technologies were low, suggesting that uptake was not straightforward [31, 33]. As part of the ToC revisions process, the team used this information to rethink the pathways that linked telemedicine to other project goals and to refocus their intervention on understanding the acceptability, access and coverage of mHealth services. In the case of Bangladesh, the three surveys capturing the baseline, midline and endline information from the study area provided crucial information for implementation. The information from the midline survey helped the team to verify the assumptions in the ToC and eventually revise them to reflect the situation in the field. Accordingly, the endline information will help assess the assumptions put forward in the revised ToC and answer questions relating to how far telemedicine could link village doctors with formal health systems. The Bangladesh’ team refocus on the appropriateness of the intervention and partnerships in this context and in response to the recognition that the initial intervention was not unfolding as planned are characteristic of ‘double-loop learning’.

### India

In light of the first 2 years of implementation, the India team’s initial ToC was too broad and ambitious, based on strict timelines and assumptions. The process of ToC revision facilitated the team’s perspective to change, toward being more flexible, more mindful of the process of implementation, and more in tune with changes in the context and actors involved. During the revision, the original ToC served as a frame of reference for learning as implementation progressed, particularly around the assumptions made by the research team (e.g. about the context and stakeholders related to the project). For example, the ToC revision facilitated the India team’s reflection on several contextual changes that occurred during the second year of implementation. The most prominent of these was the change in the state leadership, which resulted in new actors leading the health and child development ministries. Based on these changes, the India team adjusted their stakeholder engagement approach in order to better understand their priorities. The ToC revision led to changes in focus of the ToC itself – from one that was ‘sector focused’ and based on ‘dissemination of outputs’, to one that was ‘people focused’ and based on ‘stakeholder engagement strategies’. The India team also saw changes to the project’s monitoring and evaluation approach. While the team maintained the same objectives, the revised ToC proposes a more holistic view of intervening to improve child health by acknowledging the child health status in the broader context of maternal and child health in addition to climate, livelihood and resilience.

After the India team’s ToC revision, there was increased focus on process indicators, such as the number of stakeholder meetings, and output indicators, such as dialogue with stakeholders and stakeholder demand for evidence. The greater level of stakeholder engagement helped the team to identify new policy priorities such as linkages between health seeking behaviour for children of the Indian Sundarbans and migration or food security. As the project was completed, the ToC will form an essential part of the process documentation and evaluation exercise, wherein the team will assess the key developments and changes in the intervention. The revisions to the team’s approach were not drastically different from the original proposed intervention, but ‘single-loop learning’ was nevertheless helpful in refining the intervention over time.

### Uganda

The Uganda team, through their application of the PAR approach, had more frequent opportunities for reflection than the other teams, through regular and systematic review meetings, both internal to the team and with external stakeholders. The Ugandan researchers planned quarterly review meetings that provided regular opportunities to reflect, discuss assumptions and refine the research and implementation design. The process of revising the ToCs, in this case, was more about summarising the learning that had taken place in the first 2 years of implementation. Additionally, the research team used the ToC to guide team reflections during their final evaluation about what worked well and what did not. The Uganda team was in a unique position relative to the other teams, as the PAR implementation approach and engagement with various stakeholders facilitated ‘double-loop learning’ throughout the duration of the project.

### Accountability

The FHS teams developed ToCs as a requirement for DFID and revised them in response to a recommendation from the FHS Consortium Advisory Group. These circumstances might point to what Valters’ termed strict top-down accountability around the ToC – with country teams being accountable to donors, who have the sole power to make funding decisions. Valters explains that one of the unintended consequences of developing ToCs under such circumstances is that the intervention pathways illustrated in the ToCs are as simple as possible, purposefully ignoring the contextual complexities and political realities under which interventions unfurl. However, this potential pitfall was avoided, because, within the FHS project, both DFID and the project management team promoted ‘learning by doing’ and facilitated ToCs playing a role in this process. DFID allowed the consortium teams sufficient flexibility to develop country-specific ToCs and to adapt their implementation plans. The project management team supported teams in documenting the process of adaptation, the ToC being one of the tools that they used. All of the three teams that revised their ToCs welcomed the suggestion to revisit the ToC, primarily because the earlier versions did not clearly depict the complex nature of the intervention and/or the context.

FHS does have a logframe, with associated monitoring indicators, which represents the primary form of accountability to the funder. However, drawing linkages between the country-level ToCs and the project-wide logframe or any other project indicators was tenuous and, therefore, the country-level ToCs had only limited use for project-wide accountability. This was a potential advantage for the consortium members, as it allowed them the flexibility to adapt, as well as to be responsive to local stakeholders, to the extent possible.

We found no evidence of the teams using the ToC process to create downward accountability loops specifically with local stakeholders and end-users. This is not necessarily a limitation – as all teams, in particular the Uganda team, did engage with local stakeholders during the various phases of implementation.

## Discussion

The use of ToCs by the FHS Research Programme Consortium was initiated as a donor requirement. Over the years, the FHS teams in Bangladesh, India and Uganda used the ToCs for communication, primarily within the research teams and occasionally by directly engaging stakeholders, as well as a vehicle for ‘learning by doing’, and less so for accountability to the donor agency. Developing and revising the ToC was a team process, characterised by group reflection and dialogue about assumptions, stakeholders and pathways for implementation. The participatory engagement of stakeholders, particularly in Bangladesh and Uganda, stimulated and catalysed changes in the intervention, which were then captured and reflected upon during the ToC revision process. For all three FHS teams, the ToC revision was generally reactive and focused more on documenting change rather than directly inducing it. FHS country teams agreed that the revision was beneficial to their projects and that the timing, around half-way through the project, coincided with the need for reflection in response changes in their environment and new learnings from what works and what does not in implementation.

In most health systems strengthening and research projects, while there are multiple ways of capturing changes in interventions (for example, through annual reviews and monthly monitoring reports), there might not be a way to bring the story of implementation together until the end of a project. The FHS experience highlights that using the ToC tool and revising it, at least once, helps to adapt and refine the intervention by systematically incorporating new learning from the initial implementation years, as well as, in some cases, feedback from local stakeholders. This process can help researchers and implementers to critically ask and reflect on the ‘so what’ question – ‘what differences does this revised assumption or understanding of causal relationships have for the project?’ and to synthesise and capture learning from early implementation that would otherwise be scattered across multiple sources of information.

Although the ToC diagram and narrative provide an accessible and comprehensible way of presenting the intervention to various audiences [3], the FHS experience confirms the relevance of the ToC development and revision process, which goes beyond the product itself. For example, a colleague from Uganda reflected that the ToC is the tip of the iceberg: what becomes a part of the ToC is a crystallisation of many prior discussions, questioning and mulling over experiences of implementation. Therefore, the ToC process can facilitate critical thinking and learning, allowing those who take part to focus less on the micro-details of documentation. The FHS experience in this regard is consistent with the idea that ToCs could be most useful as a “*compass for helping us find our way through the fog of complex systems, discovering the path as we go along*”, as Green, quoted by Valters suggests [27]. The FHS experience confirms that the ToC process can facilitate ‘second order’ or ‘double-loop’ learning, which is not about getting closer to the ‘truth’ (the ‘correct’ system or ‘correct’ assumptions), but about an iterative process through which there are shifts in organisational outlook, ways of working and the pathways of change; the context which influences change, as well as both successes and failures in implementation, are critically assessed [3, 30].

The FHS experience contributes insights to outstanding debates around the development of ToCs, the timing of revising them, the type of evidence needed and the engagement of stakeholders. On the development and revision of ToCs, FHS teams have remarked that periodic special meetings or periods of reflections are important, as revisions to the intervention are constantly happening during implementation, sometimes too often to be immediately captured in the ToC. Additionally, especially when managing a large-scale intervention with many components, it is difficult for all the relevant information and changes that may happen to be captured in one ToC.

Implementers and researchers alike also wonder how much evidence they would need to develop ToCs and how rigorous the process should be. Valters, for example, argues that ToCs be approached as a way of working, rather than static “*evidence document*” – meaning that, even in the absence of evidence, it can be useful [3]. For example, the ToC can be based on weak and selective evidence. It can also be difficult to identify all key assumptions in advance of implementation and which assumptions to include within the ToC. While included assumptions should be ones that are critical to the ToC, in practice, there are many critical assumptions (the government remains stable, the donor continues to fund) and not all can be included. In this case, the ToC can identify pathways that need to be interrogated further, and revised as further information becomes available. A potential pitfall of the ToC tool is that it can promote linear thinking by reinforcing the impression that outcomes of interventions can be predicted [3] or by using a ToC solely to check whether implementation plans are “*true to their original intent*” [19]. Certainly, the FHS teams’ revised ToCs appear less linear than the early ones. FHS experience demonstrates that multiple information streams and engagement approaches can be used to inform ToC development and revision. Each country used slightly different approaches, likely signaling that the type and level of evidence needed should be assessed on a case by case basis.

Further, users of ToCs often debate about whether, when and how to incorporate stakeholder perspectives. The FHS experience suggests that, ideally, stakeholders should be engaged early and consistently, if possible, though not necessarily just through the ToC processes. In Uganda, for example, incorporating stakeholder perspectives at the beginning while planning and designing the project was very useful because it helped the project to start off with more realistic expectations or assumptions. The PAR approach then facilitated systematically obtaining stakeholders’ perspectives and feedback.

Finally, concerns persist around the role of donors in ToC development and how to manage donor expectations and communications about changes and revisions. Because ToCs are increasingly mandated by donors, implementers could develop ToCs simply to check off a box, without intention to use or revisit it during implementation. In such cases, many of the potential benefits of ToCs could be missed. Further, because of pressure to demonstrate results, implementers may feel disinclined to use the ToC honestly to document critical challenges faced during implementation. Therefore, according to the literature, and to FHS experiences, ToC processes are best kept flexible, with a focus on the ‘ToC thinking’, as a reflective approach to think through issues. Although FHS was required to develop ToCs by the donor, the country teams had little pressure to use the ToC for accountability for results, or to update and review the ToC as implementation progressed.

Our discussion and conclusions based on the FHS country team experiences are limited by the fact that the ToC process was not systematically documented in real time and we relied on our collective, retrospective recollections, which are incomplete and not entirely systematic.

## Conclusions

The ToC development and revision processes have been useful for FHS, helping the research teams, and occasionally key local stakeholders, to make their assumptions and mental models about their respective interventions explicit and to learn from implementation. Revising the ToCs fostered recognition of the adaptive nature of health systems and emphasised the time, space and flexibility that health systems strengthening programmes must have in order to cope and thrive in such complex adaptive systems. Future projects using the ToC approach should ensure that their focus is on reaping the benefits arising from the process of developing the ToCs, rather than on the ToC visual depiction and narrative. Furthermore, they should continue to reflect on the use of ToCs, specifically to foster ‘double-loop learning’. Organisational learning remains a ‘black box’ [2] and it certainly requires more than the ToC tool. Nevertheless, incentivising the use and revision of ToCs during project implementation, as well as further documentation of how ToCs are used in practice, could reveal additional ways in which this tool and associated process could facilitate learning about intervening in complex systems.

## Open peer review

Peer review reports for this article are available in Additional file 2.

## Additional files

Additional file 1: Figures of country Theory of Change diagrams. (DOCX 1673 kb)

Additional file 2: Open peer review reports. (DOCX 41 kb)
