# Supplementary material for: Using Theories of Change to inform implementation of health systems research and innovation: experiences of Future Health Systems consortium partners in Bangladesh, India and Uganda
Source: Health Res Policy Syst. 2017 Dec 28;15(Suppl 2):109. doi: 10.1186/s12961-017-0272-y (PMC5751673; doi:10.1186/s12961-017-0272-y)

Figures

1. Original ToC, Bangladesh team


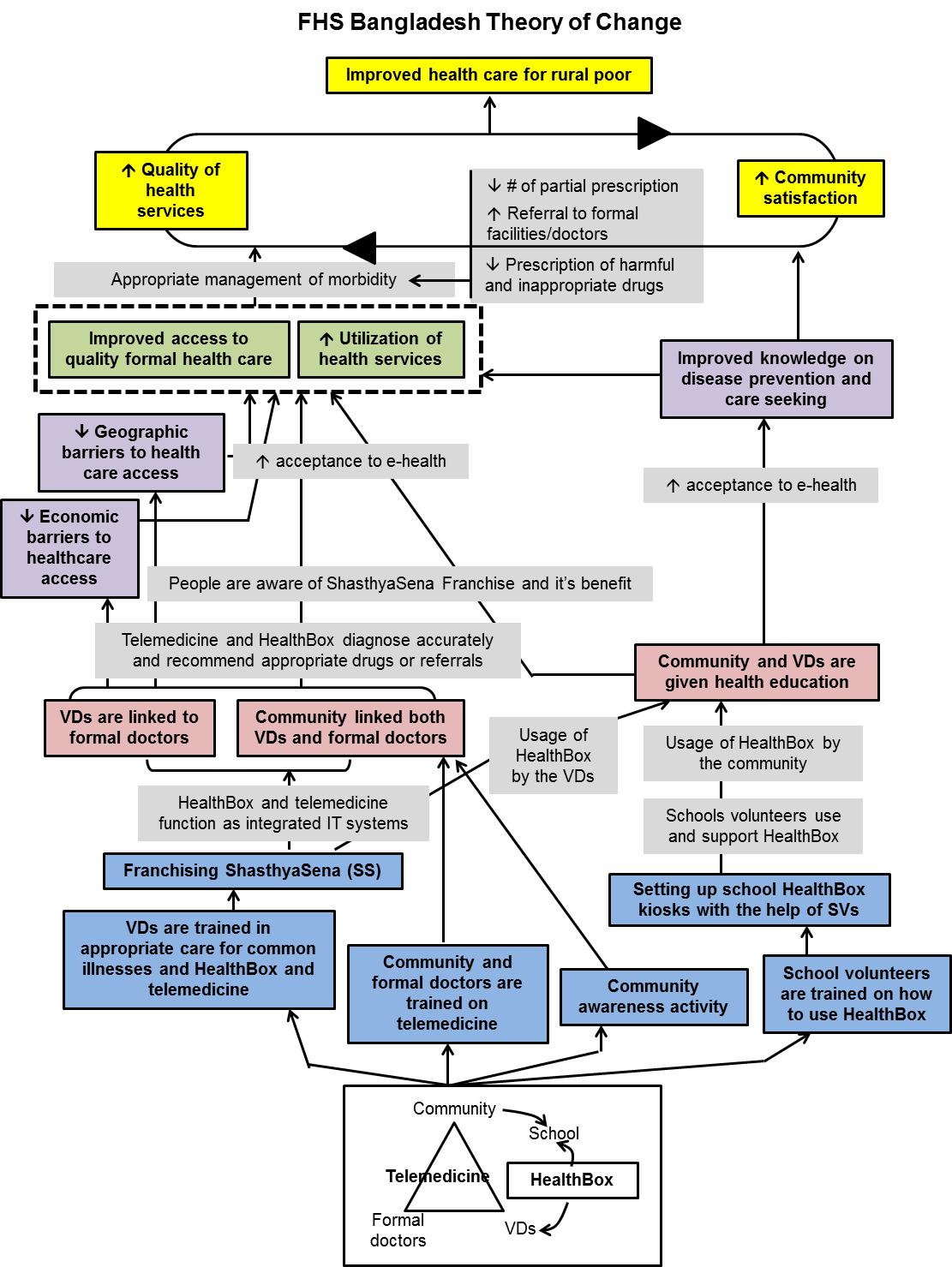

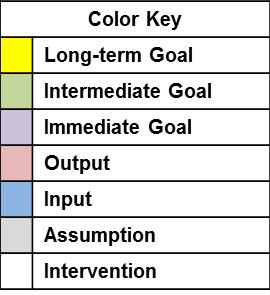


1. Revised ToC, Bangladesh


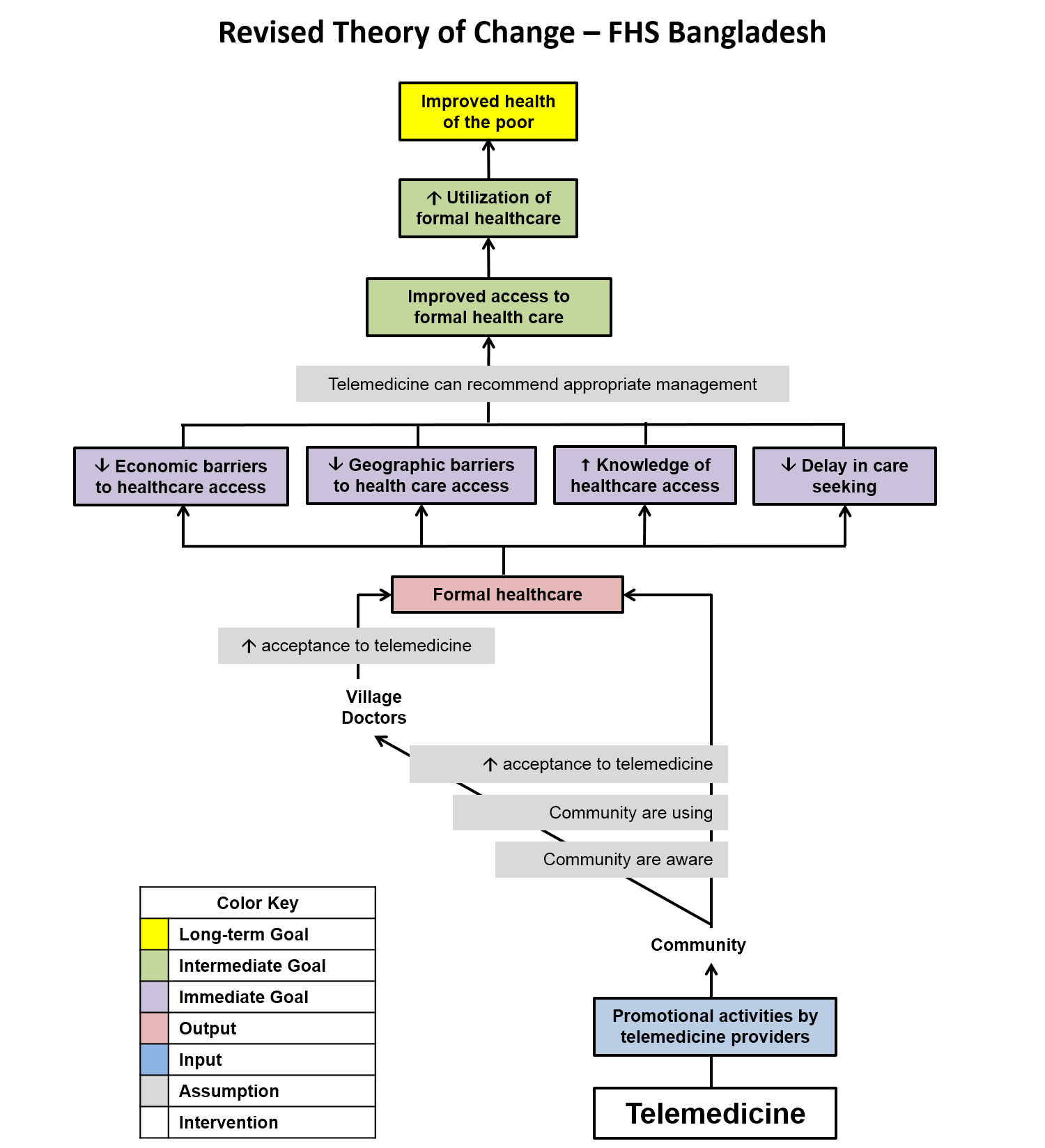


1. Original ToC, India team
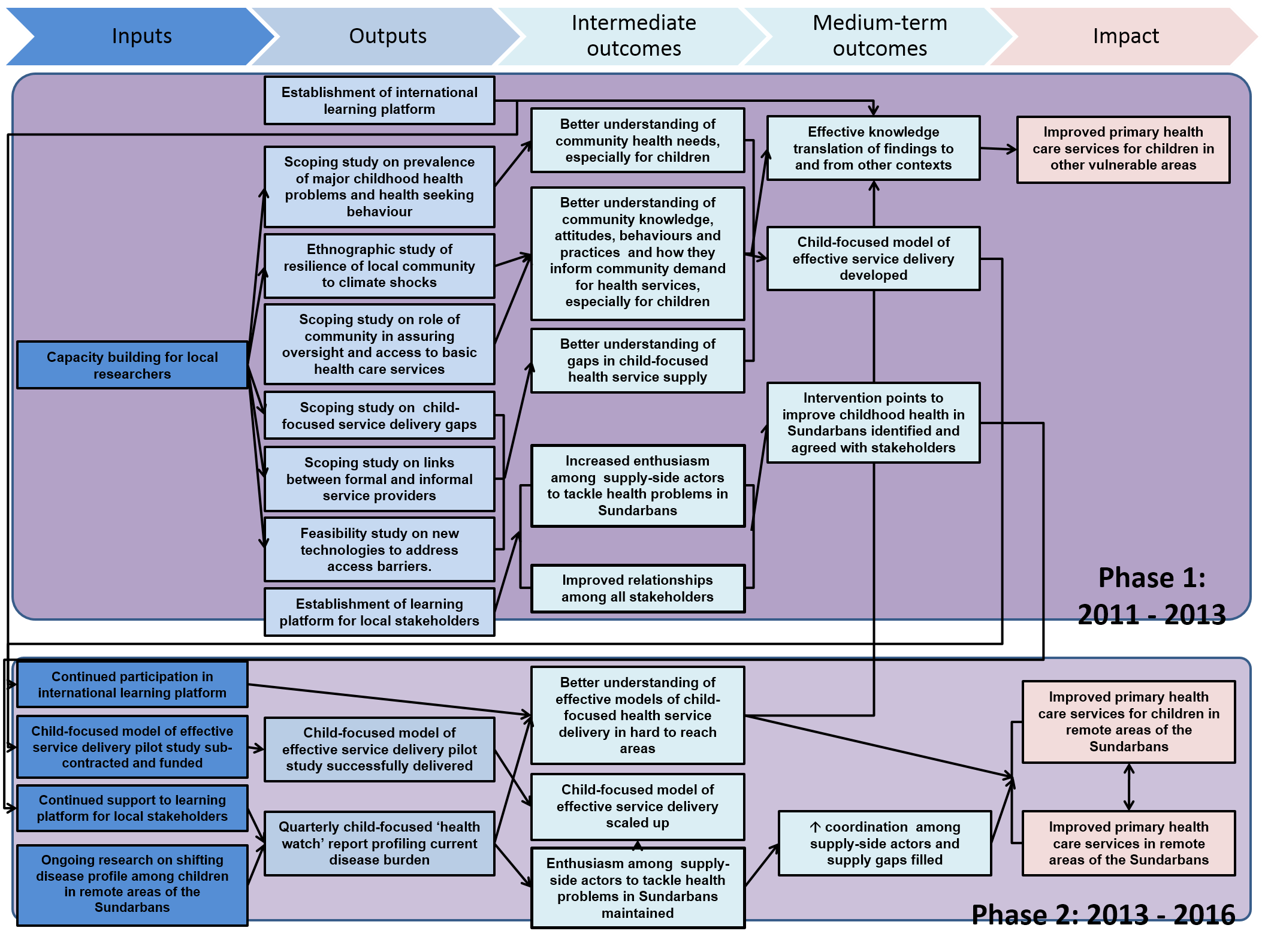

2.
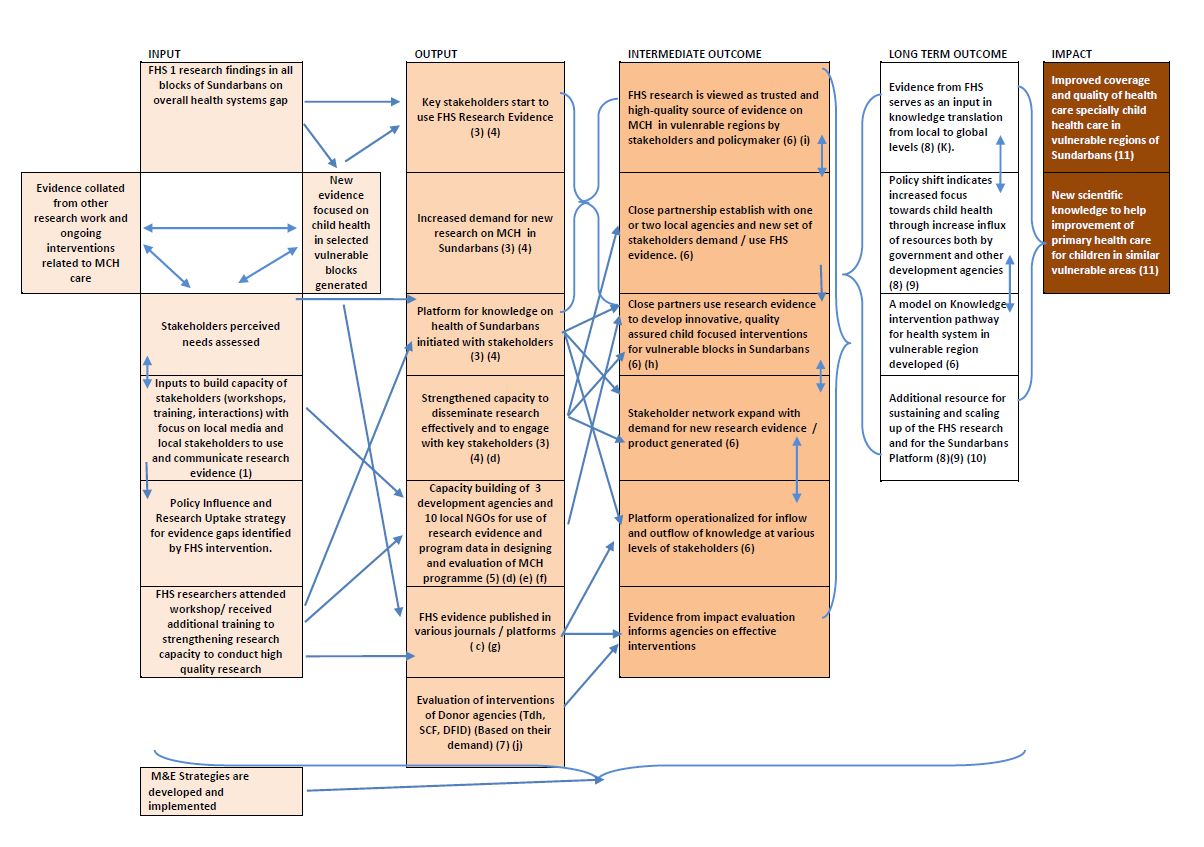
Revised ToC, India team
3. Original ToC, Uganda

1. Revised ToC, Uganda


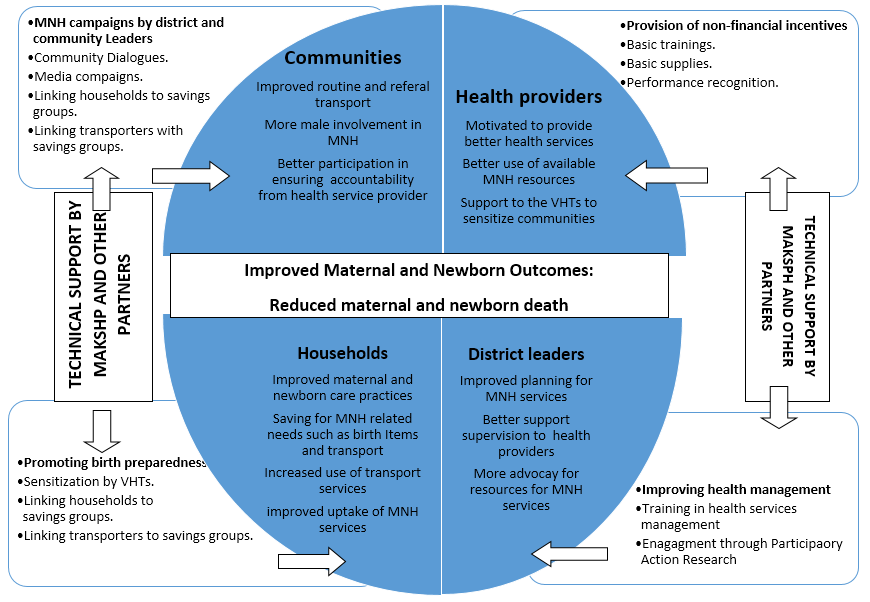


E. FHS Consortium wide Theory of Change


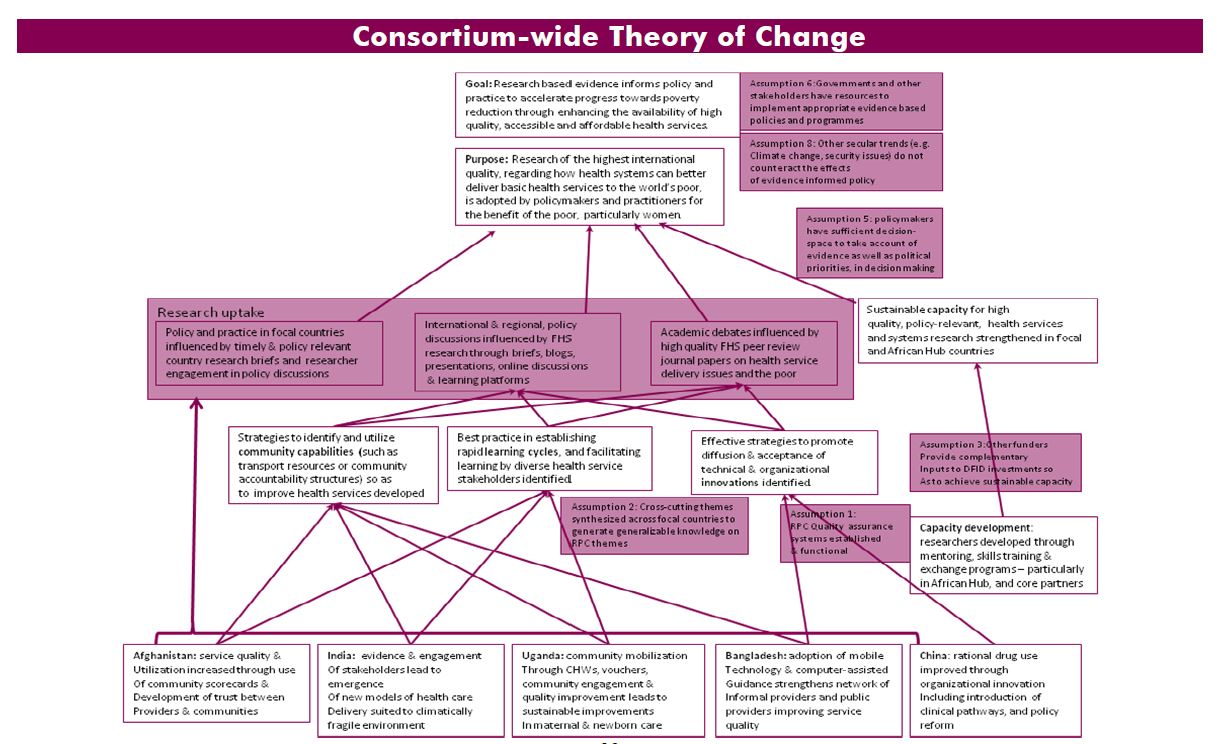

Supplement: Supplementary file 1 — Figures of country Theory of Change diagrams. (DOCX 1673 kb) [file 12961_2017_272_MOESM1_ESM.docx]
