# Supplementary material for: Using Theories of Change to inform implementation of health systems research and innovation: experiences of Future Health Systems consortium partners in Bangladesh, India and Uganda
Source: Health Res Policy Syst. 2017 Dec 28;15(Suppl 2):109. doi: 10.1186/s12961-017-0272-y (PMC5751673; doi:10.1186/s12961-017-0272-y)
Supplement: Supplementary file 2 — Open peer review reports. (DOCX 41 kb) [file 12961_2017_272_MOESM2_ESM.docx]

**Title**: Using Theories of Change to inform implementation of health systems research and innovation: experiences of Future Health Systems consortium partners in Bangladesh, India, and Uganda

**Reviewer 1:** Gill Schierhout

**Reviewer’s report**

**General comments**

This paper draws on the experiences of a donor funded research consortium in using theories of change. It describes the approach to Theory of Change (ToC) development and revision by project implementing teams in three of five member countries. The paper addresses an important topic, and the methods appear generally robust. The discussion section in particular contains some useful insights. There are however, in my view, a number of weaknesses in the manuscript that need to be addressed to bring out the key messages of the paper and to meet publication standards. I have made some suggestions below. I hope that the authors find these useful and constructive.

**Major revisions required**

1. Length and coherence: While I appreciate the article is for a journal supplement, I feel that at 7000 words excluding tables and references, the paper is unnecessarily lengthy and should be made more concise. The substance of the article does not seem to be sufficient to justify its current length, and I was struck with a number of instances of de ja vu when reading. The background section does not seem to lead into the methods and findings, and neither leads well into the discussion section (which is the strongest part). I strongly recommend the authors consider how to tighten the paper. I tentatively made some suggestions below, but the authors may have better ideas.
2. Clarity of intent - It is not clear to me who the paper is targeted at, and what the authors would like the target audience to do differently. For example, is the paper recommending that development agencies require (or not require) ToC revision – and if so, what are some of the key lessons from the authors’ experiences, to make the use of this tool most productive? I think this is mostly there, but somewhat hidden and this reader found the key messages of the paper difficult to discern. The authors undoubtedly have much valuable experience from their work in this area, but the current version of the paper does not, to my mind, do this justice. As a result the overall conclusion appears somewhat weaker than it needs to be.

**Title**

The title is clear. However much of the paper’s findings seem to focus on ToC revision, rather than the broader issue of ‘use’. Indeed, the justification provided for excluding two countries from the analysis was that ‘further revision of their ToCs was not useful at this point’ (p9, line 7). Please consider whether the title is correct as it is, or whether it would be more accurate to focus the title on ‘revision’ of ToC.

**Abstract**

The abstract at 408 words, is longer than the maximum of 350. The conclusion of the abstract comes across as rather weak. I recommend that the authors re-visit the abstract after addressing other revisions to the paper.

**Background**

**Major revision required**

1. I found the Background a bit rambling and difficult to follow. I suggest the authors consider how to make the central message of this section much clearer and cut some of the distracting detail. For example, the authors state that there has been a rapid growth of interest in Toc (p4 line 4), and later (p5, line 9) refer to ToC as ‘re-emerging’ in the realist evaluation literature. I was left wondering how the emergence, then wane of interest, and then re-emergence in ToC is relevant to the topic of this paper, which is essentially cross-sectional. Similarly the description about different ways in which the ToC has been addressed by different bodies of literature does not seem entirely relevant, or if it is, the relevance of this is not made clear. These seem to me to be side-issues that distract from the central message that the Background section is driving to. It seems to me that the central message of the Background section is that the ToC approach is used a lot, and while ‘process’ is central to the approach, there has been little documentation about ‘process’ and what it entails – so this is the gap the paper is trying to fill.
2. Relatedly I wondered if some of the literature in the background would fit better in the Findings section, where there would be opportunity to critically reflect on the literature in the light of the experience of the consortium.
3. Objectives of the paper: I think the reader would be guided through the intent of the paper much more easily, if the authors clearly specify a couple of objectives of the paper, and make sure these are met. There are a range of intended objectives of the paper alluded to in the Background section (page 7, lines 2-13). I am not convinced that all of these are fully realised, or that what is promised in these lines has been delivered. For example the authors state that, “we conclude with practical reflections and lessons learned on the usefulness of ToCs and their role in the broader array of tools and approaches to facilitate flexible implementation and learning by doing”. (p7, lines 10-13). But the reader gets to the end of the paper without a clear message about what can be learnt about how the ToC fits other tools and approaches to facilitate flexible implementation – or indeed, what these tools and approaches are. Is there a conclusion the authors could draw for example, on the way that the consortium included use of both the logframe and ToC? Do the authors feel this was confusing and led to duplication of effort, and donors should choose, or are these tools complementary in the experience of this project, and if so, how? Similarly for Participatory Action Research as used in Uganda – was this complementary or duplicative? Do you need both? The purposes of PAR, ToC and logframes all have some overlap it seems – could this be defined more clearly (perhaps as a sub-section in the background), and then in the findings and conclusions section, observations made about how this played out in practice in this project? These things are alluded to, but this reader in any case found the messages and conclusions difficult to discern.

**Minor revisions required**

1. The referencing formatting needs attention as the numbering does not appear correctly in the text (p6, line 11; p7, line 9).
2. Please consider using sub-headings to make the material easier to follow.

**Methods**

**Minor revisions required**

1. The methods are generally clearly described, although it would be helpful to have a little more detail about the people who participated in the reflection discussions, their roles, length of time in the project, and so on, if this is available. This is after all the main data on which the findings are based.
2. It seems to me that since the main findings of the paper relate to revision of the ToC in 3 out of the 5 countries in the consortium (p8, line 20 refers), and indeed this is promised in the title. The selection of these 3/5 countries needs to be made clear in the methods – perhaps use an abbreviated version of the text on p9, line 4-9?). As it currently stands, the reference to 5 countries in the Findings section is somewhat confusing.
3. It surprised me that the methods did not include a review of the project M&E documentation - I would have expected the ToC to be used for M&E, and the paper promises to describe use of the ToC. Please clarify.
4. The authors mention a ‘focused, critical review of the peer-reviewed and grey literature’ (p7, lines 20-21). I was then expecting to find some mention of what was found from use of this method in the Findings, but there is none. From p7, line 21-22, ‘examples of how the ToCs were used, similarly to the experience of the FHS project’ – but no examples are presented in the findings. I think this review should either be removed from the methods or the findings of the review presented clearly in the findings section of the paper.

**Findings**

**Major revisions required**

1. P8, line 17-12: As discussed above, there are no data presented for Afghanistan and China – the exclusion of these countries should be concisely explained in the methods. The information that it the ToC construction was mandatory is repeated in a number of places elsewhere (e.g. p10, lines 19-21; p19, lines 19-20).
2. The findings section overall has the feeling of ‘raw data’ and needs a bit more synthesis and focus in line with the objectives of the paper. I was not convinced by how the material was structured within the four potential ‘uses’ of ToC, and think this needs more consideration (points below refer).
3. P10, lines 21-p11, page 11, lines 1-19 – this long discursive on the logframe does not to my mind sit well under the subheading ‘strategic planning’. The information provided from p11, lines 20-22 seem more relevant to the use of ToC in ‘strategic planning’. Please consider the the best place for the discussion about the logframe and ToC (noting it is raised again in relation to accountability – perhaps that is a better fit?).
4. P12 2-p15, line 17: communication – please consider the use of sub-headings here – the text seems to jump around from internal to external and back to internal communication and it is hard to follow the logic. I am not left with a clear picture of the use of the ToC revision in facilitating communication. Text on p13 lines 3-6 seems more about use of the ToC for ‘learning’ rather than for ‘communication’.
5. P16, line 10-p19, line 17 – this is interesting granular detail and provides an overview of the process of revision of the ToC for all 3 countries (it is not just about use of the ToC for learning, as indicated by the title). One solution to the length of the paper is to describe the process of revision of the ToC in each country (basically move this text, or some version of it, to come first in the findings section), and then, once the clear picture is established of exactly what was done, have a much tighter summary of use of ToC in relation to learning, communication, accountability and strategic planning (e.g. p21 lines 2-19, and others). There could then be a much shorter tighter discussion that focuses on drawing the paper to a conclusion. This is offered as one possible solution – but there may be others. The main point being that there is just too much jumping about in the paper in its current form, and duplication between the different sections needs to be removed.

**Minor revisions required**

1. Table 1: The information in this table might be more economically presented in a box. Since the title of the paper refers to ‘health systems research and innovation’, I wondered how the intervention foci described here reIate to health systems research. This may need some explanation. What is the intervention and what is the research?
2. Table 2: this promises a summary of the projects and process of developing and revising the ToC.
   a. The quality of editing of Table 2 is rather weak.
   b. The row ‘major contextual changes captured’ is difficult to understand. Captured by what? By the ToC revision? By the ToC itself? By the project as a whole? Further, the text does not relate to the title - for example, the text for ‘Uganda’ does not seem to be a contextual change captured by the ToC, but rather seems to describe some of the changes in the project itself. Similar comments could be applied to some of the text in this row for the other countries.
   c. The table could be shortened by keeping only the key messages that the authors want to convey. For example, the detail about MANIFEST and MANEST is not mentioned elsewhere in the article, and I question whether it is adding to the authors’ purpose for this table.
3. P10, lines 1-16. This is interesting text about how the revised ToC differed from the original ToC in each case. This seems to relate to Table 2, and perhaps some cross-referencing would help the reader here to see the linkages. It is hard not to be left with the impression that in some cases the revised ToC process was reactive rather than proactive – that is, it was just about documenting change, rather than using the ToC revision process to catalyse change. Perhaps I have got this wrong. Can the authors say anything more about this?
4. P8 and 9 – ‘revising’ and ‘revisiting’ seem to be used interchangeably. Was this intended? One may revisit without revising.
5. P13, lines 7-12 – please edit to the active voice.
6. P13, line 14 – this is the first mention of the participatory research approach. It is discussed again in more detail later. Perhaps add a reference or short definition for those unfamiliar with the approach so that implications of PAR can be appreciated. The impression left is perhaps the ToC was superfluous to communication in Uganda, since the team were using a PAR approach for the project. Is this correct? Please clarify. It is difficult to tease out from the description on p13, lines 14-24, what the role of ToC revision was, if any, in all of these activities. Relatedly, text on p15, 9-17 discusses PAR again in relation to communication but no clear point is made.
7. P15, line 5 – please edit to the active voice
8. P16, 1-10 – are the authors suggesting that some experience with implementation is necessary to develop a robust ToC?
9. P17, line 2 – please check – ‘provoking’ or ‘providing’?
10. P18, line 20 – please reword – ‘to uptake the research findings’
11. P20 – line 4 – can the authors provide information on what the “incentives necessary for the ToC to be part of the process” were. This seemed to be the first mention of incentives.
12. P20 line 14-15 – consider rephrasing or taking out the phrase “hinting that ToCs are better applied to interventions and that they are more easily developed the closer they are to the intervention context” – it is not clear what this means and what logic it is based on.

**Discussion**

**Minor revisions required**

I think this should be much tighter and refer back to the objectives of the paper.

1. P22, lines 23-24 – Do the findings of this project support the assertion that “ToCs are most effective when integrated… as opposed to when they are mandatory.” My understanding is that these ToCs were mandatory. Some additional reflection or comment is needed.
2. P23, line 12-23 – can the authors drive this to some conclusion. It is not clear has been learnt about this issue.
3. P24 – are there other limitations that need to be mentioned?

In some cases the project experience with ToC gets a bit lost, or merges into the general project experience, and overall for this to work as a paper, I feel that the ToC contribution, and the contribution particularly of the revision of the ToC, needs to be more clearly drawn out.

**Declaration of competing interests**

I declare that I have no competing interests.

**Reviewer 2:** Erica Breuer

**General comments**

In general this was a well-written article on a topic which has not yet been covered by the ToC literature.

It seemed quite long and I think making the paper more concise and focusing on the key findings would strengthen it. The authors also used a lot of quotes from the literature which I think could be paraphrased to make the paper more concise. In some cases it is not clear why authors use parentheses are used, for example “Although the ToC diagram and narrative are able to evoke the “story” – an accessible and comprehensible way of presenting the intervention to various audiences…”

The methods are clearly stated and although it is not possible to replicate the work, the authors provide enough detail for a reader to make a judgement in relation to the credibility of the evidence presented. The paper is descriptive and describes the use of ToC by the FHS consortium but does not make of any formal data analysis techniques.

The discussion and conclusions were interesting and put the paper into context in terms of the wider field.

The abstract summarises the paper well.

I particularly enjoyed the health systems focus and the focus on ToC thinking and the reflection of the Ugandan colleague about the tip of the iceberg.

**Major compulsory revisions**

1. The authors claim to be answering quite a number of research questions, namely,
a. to understand the circumstances and rationales under which ToCs were applied and whether or how ToCs can facilitate iterative design and learning, as well as the engagement of key stakeholders.
b. to shed light on some of the outstanding debates about ToCs: when should ToCs be developed and whether and how they should be revised, what level of evidence is needed to develop a ToC, and when and how to incorporate stakeholders’ perspectives?

Although the authors did answer the first set of questions ( a.) I did not feel like question b. was explicitly answered. I think this is partly because the findings are structured according to Valter’s framework. I also think it is because the data available and the experience of the FHS consortium does not actually lend itself to answering questions about stakeholder involvement as they did not seem to be actively engaged in the ToC development in most cases. I think the authors could consider more carefully how to structure their results in order to make the answers to the above questions more explicit. The stakeholder issue was mentioned and discussed in the results.

2. Using the Valter’s structure of strategic planning, communication, learning and accountability was helpful to frame the findings. I have some comments in relation to these sections:

In relation to the strategic planning section, this focused primarily on the relationship between the consortium ToC and logframe and country ToCs (which seems to belong more in the accountability section than the strategic planning section). There was no real discussion on HOW each country partner used the ToC for planning within their context. For example, was it the only planning tool they use or did they use other frameworks or priority setting exercises. What was the potential advantage of ToC over other types of management or planning tools?

Communication: I found this section to be quite long and discursive. I understand that a lot of complexity needs to be conveyed but I think it would be helpful if the authors focus on the key findings they want to present with examples rather than trying to describe all the ToC process in each of the countries.

Accountability: I felt the authors took a rather narrow view of accountability here. There is once again discussion of the consortium ToC but no real description of how the country ToCs were substantiated by the work of the project. For example, did each country have indicators to measure the short, medium or long term outcomes of their work. If not, how did they know whether or not the change in the ToCs was actually happening? Or was the activity measured at the output level only? To some extent this was described in the section on learning but it would be interesting to know whether indicators to measure the success of the ToC were measured at the country level.

For completeness, it may be helpful for the reader to view the consortium ToC so that (s)he can appreciate the difference between the country level and consortium ToC.

3. The authors refer to the assumptions of the ToC but these are not included in the ToC diagram in India and Bangladesh. Neither are some other elements such as stakeholders, donors, etc.

4. The authors state, “For example, the ToC can be based on weak and selective evidence.” It is not clear from the description of the ToC processes what evidence was used to inform the development of their ToCs.

**Minor revisions**

1. Table 2 has COORDINATOR written in CAPS. It would be helpful to include how the original ToCs were developed (face to face, via email, using literature, using programme experience, interviews, etc.)

**Declaration of competing interests**

I declare that I have no competing interests.

**Author’s response to reviews**

Reviewer 1: Gill Schierhout

**Overall**

**Major revisions required**

3. Length and coherence: While I appreciate the article is for a journal supplement, I feel that at 7000 words excluding tables and references, the paper is unnecessarily lengthy and should be made more concise. The substance of the article does not seem to be sufficient to justify its current length, and I was struck with a number of instances of de ja vu when reading. The background section does not seem to lead into the methods and findings, and neither leads well into the discussion section (which is the strongest part). I strongly recommend the authors consider how to tighten the paper. I tentatively made some suggestions below, but the authors may have better ideas.

*Thank you for your useful suggestions. We tried to address as many suggestions as possible (see below for detailed notes). While we recognize that the paper is on the longer side, we would like to propose that we do not restructure the paper at this time. Instead, we have revisited the flow of the paper and eliminated redundancy. We also ensured that the transition between sections is clearer.*

4. Clarity of intent - It is not clear to me who the paper is targeted at, and what the authors would like the target audience to do differently. For example, is the paper recommending that development agencies require (or not require) ToC revision – and if so, what are some of the key lessons from the authors’ experiences, to make the use of this tool most productive? I think this is mostly there, but somewhat hidden and this reader found the key messages of the paper difficult to discern. The authors undoubtedly have much valuable experience from their work in this area, but the current version of the paper does not, to my mind, do this justice. As a result the overall conclusion appears somewhat weaker than it needs to be.

*Thank you for these important observations. Based solely on the FHS experience we cannot recommend that donor agencies require ToCs or not. We would like to make broader statements about how ToCs could be used and the types of questions that can be answered in the process. That said, we do recognize that our conclusions need to be clearer and added a conclusion section. Furthermore, we have made edits to the paper objectives and have hopefully clarified our scope and audience.*

**Title**

The title is clear. However much of the paper’s findings seem to focus on ToC revision, rather than the broader issue of ‘use’. Indeed, the justification provided for excluding two countries from the analysis was that ‘further revision of their ToCs was not useful at this point’ (p9, line 7). Please consider whether the title is correct as it is, or whether it would be more accurate to focus the title on ‘revision’ of ToC.

*Thank you for pointing this out. We had interpreted “use” of the ToC to also include revision. We chose to keep the title as is, so as to not imply that the paper would focus only on the revision process or on the revised TOC.*

**Abstract**

The abstract at 408 words, is longer than the maximum of 350. The conclusion of the abstract comes across as rather weak. I recommend that the authors re-visit the abstract after addressing other revisions to the paper.

*We have edited the abstract to fit the journal requirements, as well as to strengthen the conclusion.*

**Background**

**Major revision required**

4. I found the Background a bit rambling and difficult to follow. I suggest the authors consider how to make the central message of this section much clearer and cut some of the distracting detail. For example, the authors state that there has been a rapid growth of interest in Toc (p4 line 4), and later (p5, line 9) refer to ToC as ‘re-emerging’ in the realist evaluation literature. I was left wondering how the emergence, then wane of interest, and then re-emergence in ToC is relevant to the topic of this paper, which is essentially cross-sectional. Similarly the description about different ways in which the ToC has been addressed by different bodies of literature does not seem entirely relevant, or if it is, the relevance of this is not made clear. These seem to me to be side-issues that distract from the central message that the Background section is driving to. It seems to me that the central message of the Background section is that the ToC approach is used a lot, and while ‘process’ is central to the approach, there has been little documentation about ‘process’ and what it entails – so this is the gap the paper is trying to fill.

*Thank you for these suggestions. We have examined the background section once more very carefully to make sure that the various threads are easy to follow. The background section has 4 paragraphs, on the following topics: 1) what are TOCs; 2) history of TOCs; 3) re-emergence of TOCs, recent uses, discourse and the gap in process documentation; 4) introduction to FHS and the paper objectives. We clarified the language around objectives, essentially to focus on 1) documenting the process of TOC development/revision using the FHS experience and 2) adding to current debates on when they should be developed etc. We moved around and/or deleted some of the other text so that it does not draw attention away from the objectives. The material covered in the deleted text is moved to the methods and results.*

5. Relatedly I wondered if some of the literature in the background would fit better in the Findings section, where there would be opportunity to critically reflect on the literature in the light of the experience of the consortium.

*While this is an interesting proposal, we would like to suggest that we do not make this move. We intended the literature review to help us write the background to TOCs and do not have much more to report than what is presented in the introduction. To avoid misunderstandings, we have deleted the literature review from the methods (as recommended below), and focus on the findings section on the documentation piece.*

6. Objectives of the paper: I think the reader would be guided through the intent of the paper much more easily, if the authors clearly specify a couple of objectives of the paper, and make sure these are met. There are a range of intended objectives of the paper alluded to in the Background section (page 7, lines 2-13). I am not convinced that all of these are fully realised, or that what is promised in these lines has been delivered. For example the authors state that, “we conclude with practical reflections and lessons learned on the usefulness of ToCs and their role in the broader array of tools and approaches to facilitate flexible implementation and learning by doing”. (p7, lines 10-13). But the reader gets to the end of the paper without a clear message about what can be learnt about how the ToC fits other tools and approaches to facilitate flexible implementation – or indeed, what these tools and approaches are.

*Thank you for these important reflections. As mentioned in our reply to comment #1, we have sharpened the objectives and removed text that was extraneous or that we were perhaps not following up fully throughout the paper. The line that you mention on page 7 was removed.*

Is there a conclusion the authors could draw for example, on the way that the consortium included use of both the logframe and ToC? Do the authors feel this was confusing and led to duplication of effort, and donors should choose, or are these tools complementary in the experience of this project, and if so, how? Similarly for Participatory Action Research as used in Uganda – was this complementary or duplicative? Do you need both? The purposes of PAR, ToC and logframes all have some overlap it seems – could this be defined more clearly (perhaps as a sub-section in the background), and then in the findings and conclusions section, observations made about how this played out in practice in this project? These things are alluded to, but this reader in any case found the messages and conclusions difficult to discern.

*Thank you for mentioning these important questions and aspects of using TOCs alongside other tools, such as logframes or the PAR approach. Unfortunately, we cannot pursue these additional topics based on the data that we have gathered for this paper.*

**Minor revisions required**

3. The referencing formatting needs attention as the numbering does not appear correctly in the text (p6, line 11; p7, line 9).

*Thank you for pointing this out – Fixed.*

4. Please consider using sub-headings to make the material easier to follow.

*Thank you for this suggestion. We tried sub-headings, but felt that the background section became too choppy. To make the material easier to follow, we have ensured that topic sentences for each paragraph are clear. (see also answer to comment #1)*

**Methods**

**Minor revisions required**

5. The methods are generally clearly described, although it would be helpful to have a little more detail about the people who participated in the reflection discussions, their roles, length of time in the project, and so on, if this is available. This is after all the main data on which the findings are based.

*Thank you for this suggestion. I have added a bit more information to clarify the position and experience of the reflection discussion participants. (See page 7 and 8).*

6. It seems to me that since the main findings of the paper relate to revision of the ToC in 3 out of the 5 countries in the consortium (p8, line 20 refers), and indeed this is promised in the title. The selection of these 3/5 countries needs to be made clear in the methods – perhaps use an abbreviated version of the text on p9, line 4-9?). As it currently stands, the reference to 5 countries in the Findings section is somewhat confusing.

*This is a good point. We have largely moved the description that was initially in the findings section to the methods section.*

7. It surprised me that the methods did not include a review of the project M&E documentation - I would have expected the ToC to be used for M&E, and the paper promises to describe use of the ToC. Please clarify.

*The last reflection meeting, described on page 8, line 18 was intended to capture the M&E connection. We have made this more explicit in our revision.*

8. The authors mention a ‘focused, critical review of the peer-reviewed and grey literature’ (p7, lines 20-21). I was then expecting to find some mention of what was found from use of this method in the Findings, but there is none. From p7, line 21-22, ‘examples of how the ToCs were used, similarly to the experience of the FHS project’ – but no examples are presented in the findings. I think this review should either be removed from the methods or the findings of the review presented clearly in the findings section of the paper.

*Thank you for pointing out this confusion. I decided to take out the mention of the literature review from the methods. The summary of the lit review was used to write the background section and is therefore not reported in the findings.*

**Findings**

**Major revisions required**

13. P8, line 17-12: As discussed above, there are no data presented for Afghanistan and China – the exclusion of these countries should be concisely explained in the methods. The information that it the ToC construction was mandatory is repeated in a number of places elsewhere (e.g. p10, lines 19-21; p19, lines 19-20).

*This is an important suggestion and we have addressed it. The rationale for excluding Afghanistan and China has been moved to the methods. The repetition was either removed or the text was re-written to avoid sounding repetitive, if it was important to re-emphasize certain facts or events.*

14. The findings section overall has the feeling of ‘raw data’ and needs a bit more synthesis and focus in line with the objectives of the paper. I was not convinced by how the material was structured within the four potential ‘uses’ of ToC, and think this needs more consideration (points below refer).

*Thank you for this observation. We have considered internally a couple of different ways to organize the data for this paper and we feel like the four potential uses of ToCs remain the most appropriate to pursue in this case.*

15. P10, lines 21-p11, page 11, lines 1-19 – this long discursive on the logframe does not to my mind sit well under the subheading ‘strategic planning’. The information provided from p11, lines 20-22 seem more relevant to the use of ToC in ‘strategic planning’. Please consider the the best place for the discussion about the logframe and ToC (noting it is raised again in relation to accountability – perhaps that is a better fit?).

*Thank you for this observation. In this case, we feel that it is important to describe how the logframe and the ToCs were used and how they drew from one another. To help synthesize the findings under this section, we have added a summary note at the end of the section.*

16. P12 2-p15, line 17: communication – please consider the use of sub-headings here – the text seems to jump around from internal to external and back to internal communication and it is hard to follow the logic. I am not left with a clear picture of the use of the ToC revision in facilitating communication.

*Thank you for this suggestion. We have added sub-headings for each of the three areas of communication and have clarified that the last portion aims to synthesize what we learned for each country.*

Text on p13 lines 3-6 seems more about use of the ToC for ‘learning’ rather than for ‘communication’.

*We agree that this is an area also related to learning. We prefer to leave the text here as it provides a tangible example of how ToCs were used as a result of internal communications.*

17. P16, line 10-p19, line 17 – this is interesting granular detail and provides an overview of the process of revision of the ToC for all 3 countries (it is not just about use of the ToC for learning, as indicated by the title). One solution to the length of the paper is to describe the process of revision of the ToC in each country (basically move this text, or some version of it, to come first in the findings section), and then, once the clear picture is established of exactly what was done, have a much tighter summary of use of ToC in relation to learning, communication, accountability and strategic planning (e.g. p21 lines 2-19, and others). There could then be a much shorter tighter discussion that focuses on drawing the paper to a conclusion. This is offered as one possible solution – but there may be others. The main point being that there is just too much jumping about in the paper in its current form, and duplication between the different sections needs to be removed.

*Thank you for this suggestion. We have added sub-headings to make the information flow clearer. However, we feel like the specific lessons drawn are important, although they may be quite granular. As we have cleaned up and reduced the table in which the TOC is summarized (now Table 1), we hope to have reduced any duplication. We have also re-reviewed the article overall, to ensure that we eliminate any distracting changes or jumps in topic. Any leftover duplication arises from the overlap between the four dimensions we used to frame the findings. But we hope that the paper is now easier to read overall.*

**Minor revisions required**

6. Table 1: The information in this table might be more economically presented in a box. Since the title of the paper refers to ‘health systems research and innovation’, I wondered how the intervention foci described here reIate to health systems research. This may need some explanation. What is the intervention and what is the research?

*Thank you for this suggestion. We transformed Table 1 into a box, and hope that we were able to clarify the issues that you point to. We introduce each country with their research aims.*

7. Table 2: this promises a summary of the projects and process of developing and revising the ToC.
   a. The quality of editing of Table 2 is rather weak.
   b. The row ‘major contextual changes captured’ is difficult to understand. Captured by what? By the ToC revision? By the ToC itself? By the project as a whole? Further, the text does not relate to the title - for example, the text for ‘Uganda’ does not seem to be a contextual change captured by the ToC, but rather seems to describe some of the changes in the project itself. Similar comments could be applied to some of the text in this row for the other countries.
   c. The table could be shortened by keeping only the key messages that the authors want to convey. For example, the detail about MANIFEST and MANEST is not mentioned elsewhere in the article, and I question whether it is adding to the authors’ purpose for this table.

*Thank you for these observations about the table and your concrete suggestions. We have shortened and focused the text, as well as removed extraneous details.*

8. P10, lines 1-16. This is interesting text about how the revised ToC differed from the original ToC in each case. This seems to relate to Table 2, and perhaps some cross-referencing would help the reader here to see the linkages. It is hard not to be left with the impression that in some cases the revised ToC process was reactive rather than proactive – that is, it was just about documenting change, rather than using the ToC revision process to catalyse change. Perhaps I have got this wrong. Can the authors say anything more about this?

*Thank you for these suggestions. We have strengthened the linkages between the section you mention on page 10 and what is now the revised Table 1. We recognize the importance of your point on reactive vs proactive revision and reflect on it in the discussion.*

9. P8 and 9 – ‘revising’ and ‘revisiting’ seem to be used interchangeably. Was this intended? One may revisit without revising.

*You bring up a good point and an important distinction. We have checked all of the instances in which revise and revisit were used and edited for clarity, where necessary.*

10. P13, lines 7-12 – please edit to the active voice.

*Thank you for this suggestion. These edits have been made.*

18. P13, line 14 – this is the first mention of the participatory research approach. It is discussed again in more detail later. Perhaps add a reference or short definition for those unfamiliar with the approach so that implications of PAR can be appreciated.

*We added references for PAR on page 10, where it is first mentioned in relation to the Uganda activities.*

The impression left is perhaps the ToC was superfluous to communication in Uganda, since the team were using a PAR approach for the project. Is this correct? Please clarify. It is difficult to tease out from the description on p13, lines 14-24, what the role of ToC revision was, if any, in all of these activities. Relatedly, text on p15, 9-17 discusses PAR again in relation to communication but no clear point is made.

*Thank you for pointing this out. In Uganda, the TOC was not the main driver in communications with external stakeholders, but PAR was. However, the ToC was a resource which was used in other ways by the team, in support of the PAR approach.*

19. P15, line 5 – please edit to the active voice

*Completed.*

20. P16, 1-10 – are the authors suggesting that some experience with implementation is necessary to develop a robust ToC?

*Thank you for this question. We are suggesting that the learning in our case came mostly from the ToC revision rather than the ToC development. Line 10-13 on page 16 helps to clarify this point.*

21. P17, line 2 – please check – ‘provoking’ or ‘providing’?

*Thank you – providing is better suited and we made the related edits.*

22. P18, line 20 – please reword – ‘to uptake the research findings’

*Thank you, this was edited.*

23. P20 – line 4 – can the authors provide information on what the “incentives necessary for the ToC to be part of the process” were. This seemed to be the first mention of incentives.

*Thank you for pointing out this potential gap. We did not intend to start a new discussion thread at this point. The text has been edited to re-worded to remove the mention of incentives.*

24. P20 line 14-15 – consider rephrasing or taking out the phrase “hinting that ToCs are better applied to interventions and that they are more easily developed the closer they are to the intervention context” – it is not clear what this means and what logic it is based on.

*Thank you, we deleted this phase as we agree that it is insufficiently clear.*

**Discussion**

**Minor revisions required**

I think this should be much tighter and refer back to the objectives of the paper.

4. P22, lines 23-24 – Do the findings of this project support the assertion that “ToCs are most effective when integrated… as opposed to when they are mandatory.” My understanding is that these ToCs were mandatory. Some additional reflection or comment is needed.

*Thank you for pointing this out. We have reduced this sentence.*

5. P23, line 12-23 – can the authors drive this to some conclusion. It is not clear has been learnt about this issue.

*We clarified the text that you refer to.*

6. P24 – are there other limitations that need to be mentioned?

*At this time, we have no other limitations to mention.*

In some cases the project experience with ToC gets a bit lost, or merges into the general project experience, and overall for this to work as a paper, I feel that the ToC contribution, and the contribution particularly of the revision of the ToC, needs to be more clearly drawn out.

**Reviewer 2:** Erica Breuer

**General comments**

In general this was a well-written article on a topic which has not yet been covered by the ToC literature.

It seemed quite long and I think making the paper more concise and focusing on the key findings would strengthen it. The authors also used a lot of quotes from the literature which I think could be paraphrased to make the paper more concise. In some cases it is not clear why authors use parentheses are used, for example “Although the ToC diagram and narrative are able to evoke the “story” – an accessible and comprehensible way of presenting the intervention to various audiences…”

The methods are clearly stated and although it is not possible to replicate the work, the authors provide enough detail for a reader to make a judgement in relation to the credibility of the evidence presented. The paper is descriptive and describes the use of ToC by the FHS consortium but does not make of any formal data analysis techniques.

The discussion and conclusions were interesting and put the paper into context in terms of the wider field.

The abstract summarises the paper well.

I particularly enjoyed the health systems focus and the focus on ToC thinking and the reflection of the Ugandan colleague about the tip of the iceberg.

**Major compulsory revisions**

5. The authors claim to be answering quite a number of research questions, namely,
c. to understand the circumstances and rationales under which ToCs were applied and whether or how ToCs can facilitate iterative design and learning, as well as the engagement of key stakeholders.
d. to shed light on some of the outstanding debates about ToCs: when should ToCs be developed and whether and how they should be revised, what level of evidence is needed to develop a ToC, and when and how to incorporate stakeholders’ perspectives?

Although the authors did answer the first set of questions ( a.) I did not feel like question b. was explicitly answered. I think this is partly because the findings are structured according to Valter’s framework. I also think it is because the data available and the experience of the FHS consortium does not actually lend itself to answering questions about stakeholder involvement as they did not seem to be actively engaged in the ToC development in most cases. I think the authors could consider more carefully how to structure their results in order to make the answers to the above questions more explicit. The stakeholder issue was mentioned and discussed in the results.

*Thank you for these insights and suggestions. We recognized that the paper objectives needed further clarification. We have edited the last part of the introduction to clarify the paper objectives. You are right that the second objective would be difficult to meet given the information we are working with. We have therefore de-emphasized it – in the sense that we make it clear that we can reflect on these debates only to the extent that the information from FHS is available. Because we structure the findings by Valters’ framework, the discussion section will lend itself best for these reflections and we have made the necessary edits.*

6. Using the Valter’s structure of strategic planning, communication, learning and accountability was helpful to frame the findings. I have some comments in relation to these sections:

In relation to the strategic planning section, this focused primarily on the relationship between the consortium ToC and logframe and country ToCs (which seems to belong more in the accountability section than the strategic planning section). There was no real discussion on HOW each country partner used the ToC for planning within their context. For example, was it the only planning tool they use or did they use other frameworks or priority setting exercises. What was the potential advantage of ToC over other types of management or planning tools?

*Thank you for these important observations. We see the logframe and TOC as important to the strategic planning aspects of the Valters framework, though we recognize the potential overlap between the accountability and strategic planning dimensions. In a previous version we had combined them, but in the current version we do find it important to keep them separate. We have added a few sentences at the end of the paragraph to summarize the strategic planning session and reflect on the FHS country’s use of the ToC. Although it would be interesting, we are not able to add more comparative reflections at this time (i.e. TOC vs other types of management tools) as this is not something that we are able to speak to from our data.*

Communication: I found this section to be quite long and discursive. I understand that a lot of complexity needs to be conveyed but I think it would be helpful if the authors focus on the key findings they want to present with examples rather than trying to describe all the ToC process in each of the countries.

*Thank you for this observation and suggestions. We agree that this section was a bit long. We have added sub-headings to help guide the reader through our list of sub-topics and have made edits for clarification. As this was one of the principal ways in which the ToC was used by the FHS team, we did not want to lose out on too much of the details we sought to document.*

Accountability: I felt the authors took a rather narrow view of accountability here. There is once again discussion of the consortium ToC but no real description of how the country ToCs were substantiated by the work of the project. For example, did each country have indicators to measure the short, medium or long term outcomes of their work. If not, how did they know whether or not the change in the ToCs was actually happening? Or was the activity measured at the output level only? To some extent this was described in the section on learning but it would be interesting to know whether indicators to measure the success of the ToC were measured at the country level.

*Thank you for this observation. We define the accountability dimension within the Valters framework and to the extent that it applies to our data, though we appreciate that this might be limiting. As far as our documentation shows, the ToCs were not explicitly linked with project indicators. The teams used them to understand the process of implementation and relationships between various parts of the intervention system, rather to assess impact.*

For completeness, it may be helpful for the reader to view the consortium ToC so that (s)he can appreciate the difference between the country level and consortium ToC.

*Thank you for this suggestion. We have included the Consortium wide TOC among the figures, for readers’ reference.*

7. The authors refer to the assumptions of the ToC but these are not included in the ToC diagram in India and Bangladesh. Neither are some other elements such as stakeholders, donors, etc.

*Thank you for pointing this out. As mentioned in the Methods section (page 8), the narratives that go along with the TOC detail the assumptions. I added a note that these could be available upon request. They also have more information about stakeholders. In Uganda, these were more prominently part of the TOC diagram because of the application of the Participatory Action Research approach.*

8. The authors state, “For example, the ToC can be based on weak and selective evidence.” It is not clear from the description of the ToC processes what evidence was used to inform the development of their ToCs.

*Understanding the evidence that is used to develop a TOC is important. In Table 1, on page 33, we have summarized the materials that were used by each country in the TOC development and revision processes.*

**Minor revisions**

2. Table 2 has COORDINATOR written in CAPS.

*Thank you! Fixed.*

3. It would be helpful to include how the original ToCs were developed (face to face, via email, using literature, using programme experience, interviews, etc.)

*Thank you for this suggestion. In Table 1 (former Table 2), we have added more information around ToC development on in the ToC development row.*
